# Rate of obesity within a mixed-breed group of horses in Ireland and their owners’ perceptions of body condition and useability of an equine body condition scoring scale

**DOI:** 10.1186/s13620-023-00237-w

**Published:** 2023-04-06

**Authors:** Emma Golding, Ahmed Saleh Ali Al Ansari, Gila A. Sutton, Nicola Walshe, Vivienne Duggan

**Affiliations:** 1 School of Veterinary Medicine, University College Dublin, Belfield, Dublin 4, Ireland; 2 Large Animal Department, Robert H Smith Faculty of Agriculture, Food and Environmental Sciences, Koret School of Veterinary Medicine, Veterinary Teaching Hospital, The Hebrew University of Jerusalem, P.O. Box 12, 7610001 Rehovot, Israel

**Keywords:** Equine, Obesity, Body condition scoring, Owner perceptions

## Abstract

**Background:**

Equine obesity is a significant health and welfare concern. The proportion of domestic horse populations that are overweight are as high as 45%. As the primary decision-makers for their horses’ care, owners are theoretically ideally placed to identify whether their horses are appropriately conditioned, however, research in other countries has shown that many owners are unable to accurately judge their horse’s body condition. In this study, through the comparison of body condition scoring (BCS) performed by an expert and the horse owners and interviews with owners, we aimed to identify the proportion of horses that were overweight or obese, to assess the accuracy of horse owners' BCS assessment both prior to and after receiving information and instructions on body condition scoring, and to identify common themes amongst owners’ views regarding BCS assessment and the Henneke BCS system.

**Results:**

Forty-five percent of the horses in this study were overweight or obese. The agreement between the owners and an equine veterinarian regarding the horses’ BCS was fair to good both prior to (κ = .311, *P* < 0.001; ICC = .502, *P* < 0.001) and after (κ = .381, *P* < 0.001; ICC = .561, *P* < 0.001) receiving information and instructions on scoring. Three quarters of the owners who took part in the study did not use any method of monitoring their horse’s body condition. Thematic analysis of owner responses was varied, with the most common theme being an awareness of the need to monitor or make changes to their horse’s condition with responses in this theme split between owners who felt in control and those who did not. Owner feedback on the utility and useability of the scorning system was that it was useful however parts are too technical or need improvement.

**Conclusions:**

Equine obesity is a significant problem in this population in Ireland. Horse owners’ ability to accurately judge their horse’s condition does not improve with provision of instructions on body condition scoring. These results combined with owners’ feedback on the Henneke BCS system indicate that it is not a tool that can be reliably used by owners.

**Supplementary Information:**

The online version contains supplementary material available at 10.1186/s13620-023-00237-w.

## Introduction

Obesity is considered a significant health and welfare issue for horses [1, 2]. Data on the proportion of horses in Ireland that are overweight or obese is lacking however research conducted in other countries, including those with similar management practices to Ireland, suggests that significant proportion (24% to 45% or possibly as high 54.1% [3]) of their domestic horse populations are overweight or obese [4–7].

As the primary decision-maker for their horses, horse owners are theoretically ideally placed to identify health issues and seek veterinary advice early. With regards to body condition, identification of potential problems (such as increased laminitis or musculoskeletal injury risks) in a timely manner, as necessary for adequate welfare [8], may be dependent on owners being able to accurately judge how appropriate their horse’s condition is. While most research indicates that owners frequently underestimate their horses’ condition or weight [3, 5, 6], Busechian et al. (2022) found that owners may either under- or over-estimate their horses’ condition when compared to a vet’s judgement. Owners have been found to not be oblivious to the overall body condition of their horses; rather excess fat is seen as “as an indicator of health, an integral part of the horse's shape or a sign of disease” [9], sometimes as more than one of those options at any one time. It is surmised that this prevents them from recognising the presence of excess fat as a problem that requires intervention [9]. It is important to note that under-recognition of obesity is a phenomenon that has also been observed with regards to dogs by their owners [10], children by their parents [11] and even individuals by themselves [12]. Related to this phenomenon is the change in what are considered weight norms. In humans, there is evidence that the threshold at which an individual is perceived to be overweight has risen [13] while research to date indicates that horse owners are influenced by what is the norm in their environment, whether or not that norm is appropriate [14] and that people are influenced by the proposed activity of a horse when deciding whether it is underweight, overweight or appropriate [15].

There are two equine body condition scoring systems commonly used; the 9-point Henneke system [16] and the 6-point Carroll & Huntingdon system [17]. Research into the comparison of body condition scoring with other methods of non-weighing scales weight assessment, such as ultrasound [18], as well as into the applicability of morphometric measurements [5, 19] has been undertaken however they may not be similarly applicable to all breeds [5]. With regards to owners’ opinions on equine body condition scoring systems, previous research is limited, however that which has been conducted has found that owners may find the systems useful but may require training to use them [20].

In this study, we aimed to ascertain whether owners in Ireland can accurately judge their horses’ body condition, the effect on this ability of owners receiving instructions on scoring, and the proportions of horses who are underweight, appropriately conditioned, overweight and obese.

## Materials & methods

### Ethical approval

This study was exempted from full ethical review by UCD’s Human Research Ethics (approval reference LS-E-21–09-Golding-Duggan) and Animal Research Ethics (approval reference AREC-E-21–24-Duggan) Committees.

### Recruitment

A post was shared on the UCD Veterinary Hospital’s Facebook page asking for owners from counties Dublin, Kildare, Meath and Wicklow to sign up on a first-come first-served basis to a study on owners’ perceptions and decision making regarding their horse’s body condition, management, feeding, supplement use etc. Where an owner had more than one horse, they were asked to choose with which one they would like to take part.

### Assessment process

Upon arrival, the assessment process and what it would involve for both the owner and horse were explained to the owner and the owner was informed that they could cease participation at any point. Verbal consent was obtained prior to the questionnaire and clinical exams starting. One of the researchers interviewed the owner using a set questionnaire whilst the other conducted a basic examination of the horse, including assessing body condition using the Henneke body condition scoring scale. The latter researcher (referred to hereafter as the “expert”) assessing body condition was an experienced equine vet; this person was the expert for all horse assessments in this study. The exam and the expert scoring were conducted out of sight and hearing of the owner and of the researcher conducting the questionnaire portion of the visit.

### Selection of materials

During the visit, owners were provided with a two-page information sheet on the Henneke body condition scoring scale and an explanation of how to apply it to their horse (Additional file 1: Appendix 1). The Henneke system was chosen for a number of reasons; firstly, the presence of 9 scoring levels reduces the need for half scores; secondly, it consists of assessment of more discrete body areas than the Carroll & Huntingdon system which would give more areas for potential feedback from owners; and, finally, anecdotally, it is the scale more commonly used by equine veterinarians in Ireland and therefore more likely to be the one recommended or shown to clients and a “real-world” application of the research was a main aim for the researchers. The information sheet consisted of extracts from: Body Condition Scoring Horses: Step-by-Step, posted on thehorse.com on 15^th^ January 2019 by University of Kentucky College of Agriculture, Food & Environment. https://thehorse.com/164978/body-condition-scoring-horses-step-by-step/ (accessed 24/06/2021). This article was used because it: was present in the first page of results when searched using the search term “body condition scoring horses” and therefore a page that would possibly be found by owners searching for information; included the scoring chart; was concise; and, explained the process and what to look for with regards to fat build-up in the six different body areas.

### Body condition scoring - expert

Participating horses were given a basic clinical examination where all clinical parameters with the exception of temperature were checked. Where there were any parameters of concern, the owner was advised to contact their attending veterinary practitioner. Body condition was then assessed using the Henneke 1–9 scoring system and utilizing the same chart as would be provided to the owners to use. Each of the six body areas were assessed separately, and the scores totaled and divided by 6.

### Questionnaire

A researcher-administered questionnaire, consisting of both closed and open-ended questions, was conducted with each owner. The questionnaire consisted of questions covering the horse’s health history, routine healthcare, management details, and weight and condition monitoring. Owners were asked specifically if their horse had any history of laminitis or colic. Questions were asked in an open-ended manner to elicit additional comments or information from the owner. In addition to the categorical and quantitative data gathered, comments and answers to the open-ended questions from owners were also recorded; this was done by hand in spaces on the interview form. Approximately half-way through the questionnaire process, after answering questions on weight and condition monitoring, owners were shown a numerical ratingscale representing the Henneke scale complete with numbers 1–9 and descriptions of each score from “poor” to “extremely fat” (Additional file 2: Appendix 2) and asked to indicate where on the scale they considered their horse to be. They were also asked if they used any specific means of monitoring body condition and where on their horse’s body they might have noticed changes in condition. Where multiple owners from a single yard participated, this was noted to allow identification and analysis of possible trends at yard level.

### Body condition scoring - owners

After the questionnaire was complete, owners were provided with the body condition scoring instruction sheet described above. They were asked to take as much time as they needed to read the sheet and then to apply the scale to their horse whilst with the horse. Owners were free to utilize the information provided by visual and/or hands-on assessment of their horse. They were subsequently asked for the score they gave their horse as well as their answers to the following questions to obtain feedback on the body condition scoring system:

- Did you find the information on body condition scoring provided helpful?
- Will it change how you monitor your horse’s condition in the future?
- Did you find the information surprising?
- Do you feel your score of your horse has changed after considering the information?

### Statistics

Body condition scores were rounded to the closest whole number with half scores rounded up. Data were input from hard copy paper forms to Excel and analysis was performed using SPSS v.27 (IBM, 2020). Intraclass correlations (absolute-agreement, two-way random effects model, quoting single measures) were used to determine the absolute level of agreement between the expert and the owners. Cohen’s linear weighted kappa was used to assess the level of agreement taking the extent of differences between the expert and owner into account. Both tests were used for comparisons between the expert and owner scores both before the owners had received information and instructions on scoring and afterwards. Chi-square tests were used to assess association between BCS (Body Condition Score) and variables including management details, exercise routines and weight and condition monitoring. *P* values are reported with a significance level of < 0.05.

### Thematic analysis

Owner responses given in addition to their answers to the open-ended survey questions were recorded in writing and entered into a master data sheet with the responses to the closed-ended questions. Responses to questions regarding monitoring and judgment of their horse’s body condition were analysed. Initial codes were identified after familiarization with the responses. An inductive approach [21], whereby the analysis was driven by the data rather than utilising a pre-existing coding frame, was used when identifying initial codes. These initial codes were grouped into themes and these themes then applied to the owner responses, reviewed and re-named where appropriate.

## Results

### Participating owners and horses

The first 60 owners, or groups of owners where a single owner was acting on behalf of a group, who contacted the author in response to the Facebook post and whose horses were located in any of the four counties specified were included in the study. They were visited on dates between Monday 28^th^ June 2021 and Sunday 11^th^ July 2021.

The horses and ponies included in the study were of the following breeds/types: 10 cobs; nine Connemara ponies; seven Thoroughbreds; seven sport horses; four Irish Draught horses; one sport pony; one each of Selle Francais, Dutch Warmblood, English Warmblood, Holsteiner, New Forest Pony, Shetland and Welsh Cob; crosses including one Irish Draught cross, one Thoroughbred cross, one Irish Draught x Thoroughbred, one Holsteiner x ISH and one mixed native pony; and 10 with unknown, uncertain or multiple (more than two) breed heritage.

Ages ranged from three years old to one pony in its 30s (exact age unknown). Heights ranged from eight and a half hands high to 18 hands high. There were 20 mares, 39 geldings and one stallion.

Thirty-eight were kept on livery (including two DIY livery); 19 were kept privately; two were kept on livery yards but belonged to the yard owner or manager; and one was kept at a premises belonging to a friend of the owner. The number of horse-owner combinations surveyed within the same yard ranged from two to eight.

### Body condition scores

**Fig. 1:**
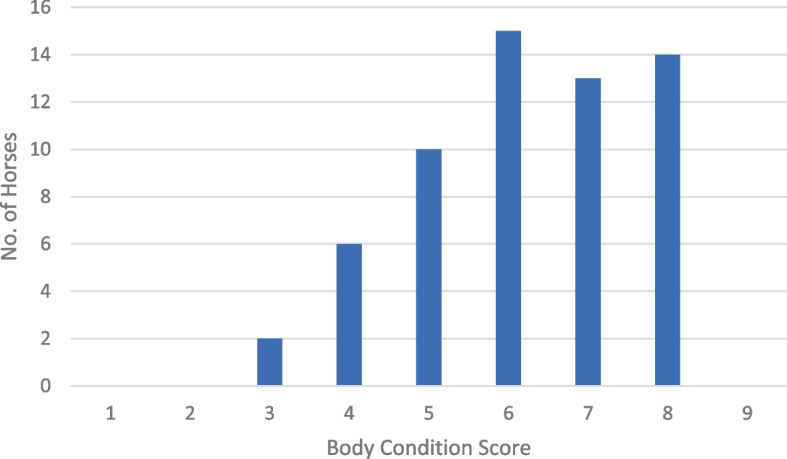
Number of horses (*n* = 60) judged to be at each body condition score

The number of horses judged by the expert to be at each of the nine body condition levels can be seen in Fig. 1. Two horses (3.33%) were under-conditioned (BCS 1–3), 51.67% were appropriately conditioned (BCS 4–6), with 10% at optimal condition (BCS 5) and 45% were over-conditioned (BCS 7–9).

No significant associations between BCS and management details, exercise routine or weight and condition monitoring were found.

### Health history

Fifty-three (88.33%) horses were clinically normal on the day of examination. One was found to have enlarged sub-mandibular lymph nodes and six had increased digital pulse(s). Seven had a history of laminitis and eight had a history of colic; of those, one had a history of both. In addition, one animal was described as being pre-laminitic and one as “a bit colicky once”. Of those animals with a history of colic and/or laminitis, five had other health issues (confirmed and suspected). Of the horses and ponies with no history of laminitis and/or colic, 23 horses had other health or behavioural issues. The confirmed and suspected health issues reported, other than laminitis and colic, included ulcers, Pituitary Pars Intermedia Dysfunction, respiratory problems, lumps, musculoskeletal problems (including arthritis), skin conditions (including sarcoids), shivers and accidental injuries however these were not explored further in this study.

### Routine healthcare

Of the 60 horses and ponies included, 49 were reported as having up-to-date flu and tetanus vaccinations. With regards to de-worming, one was reported as being de-wormed once a year; 26 twice a year; six three times a year; 10 four times a year; and one five times a year. Of the remainder, 10 were de-wormed based on FEC results; one recently de-wormed twice in quick succession due to the horse looking “ribby” and one based on consistency of the droppings; three were unspecified; and one was unknown to the owner. Fifty-one owners reported their horse being seen by an Equine Dental Technician at least once a year and 46 reported their horse being seen previously at least once by at least one allied health practitioner (these included chiropractors, physiotherapists, osteopaths etc.) in their time owned by the owner.

### Management details

Just under 50% of the horses and ponies included were living out at the time of their visits with the remainder stabled and turned out for a portion of the day or else stabled during the day and turned out overnight. Of the animals provided with forage (*n* = 43, 71.66%), 12 were provided with haylage, 29 with hay, and two with soaked hay. Forty-eight horses and ponies were given balancer and/or feed, and 37 received at least one feed supplement.

All except four horses and ponies included in the study were engaged in some exercise, ranging from occasional walking work for a 27-year-old pony to up to six sessions per week of medium (defined as a mixed session of walk, trot and canter or dressage or showjumping training) to hard (defined as a session containing a lot of canter or gallop work or cross-country jumping) work.

### Weight and condition monitoring

**Fig. 2:**
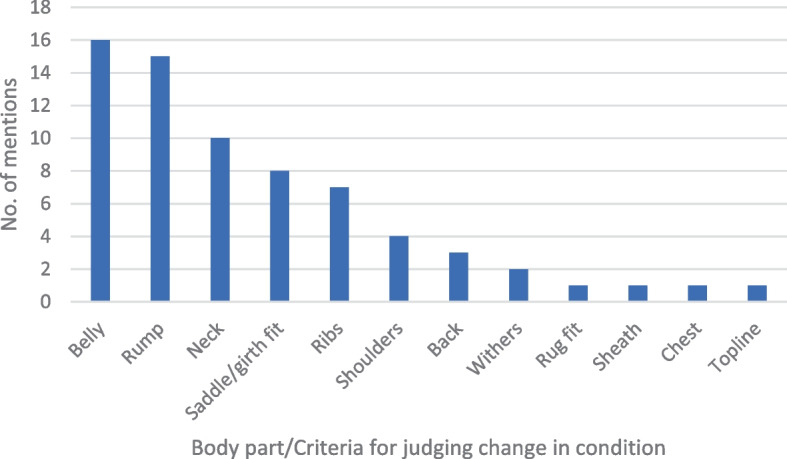
Number of times particular body parts or criteria for judging change in their horse’s condition was mentioned by owners

Six owners reported using a weight tape to monitor their horse’s weight whilst two owners reported using a specific body condition scoring system to monitor condition. One yard had a weighing scale and three of the eight owners surveyed at that yard reported using the scale to monitor their horse’s weight; four owners monitored condition by taking regular photos. Despite the majority (45) of owners reporting not using a specific method of monitoring weight or condition, of those, 37 said that they would note changes in their horse’s condition in specific areas (Fig. 2).

### Owners’ judgment of body condition

**Fig. 3:**
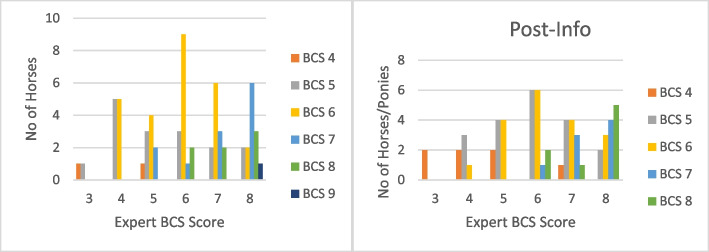
**a** Owners’ judgement of their horses’ body condition before receiving information on and  instructions re how to use the Henneke BCS system. **b** Owners’ judgement of their horses’ body condition after receiving information on and instructions re how to use the Henneke BCS system

The number of horses indicated by their owner on the numerical rating scale to be at each of the nine body condition levels, and the number of horses judged by them to be at each of the nine body condition levels after receiving information and instruction on the Henneke scoring system can be seen in Fig. 3. Prior to receiving the information about the BCS system, no owners judged their horse as underconditioned; two thirds considered their horse appropriately conditioned, with 23.33% stating that their horse was in optimal condition, and one third (33.33%) considered their horse to be over-conditioned. After receiving the information, no owners judged their horse as underconditioned; 73.33% considered their horse appropriately conditioned, with 31.67% stating their horse was in optimal condition, and 26.67% considered their horse to be over-conditioned.

**Fig. 4:**
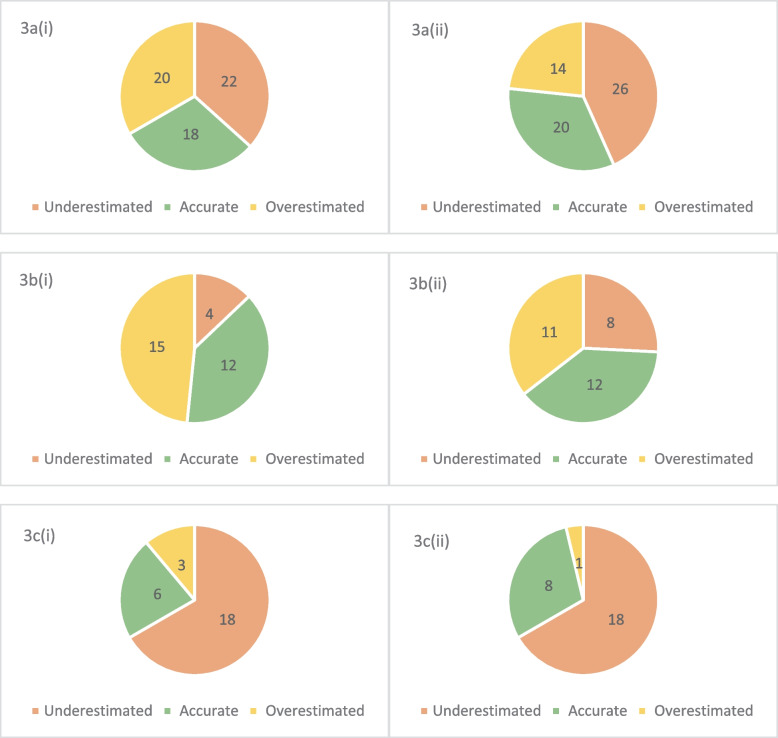
**a** (i) Owner scores before receiving information and instructions compared to expert scores for all horses (*n* = 60). **a** (ii): Owner scores after receiving information and instructions compared to expert scores for all horses (*n* = 60). **b **(i): Owner scores before receiving information and instructions compared to expert scores for horses BCS 4–6 (*n* = 31). **b **(ii): Owner scores after receiving information and instructions compared to expert scores for horses BCS 4–6 (*n* = 31). **c **(i): Owner scores before receiving information and instructions compared to expert scores for horses BCS 7–8 (*n* = 27). **c **(ii): Owner scores after receiving information and instructions compared to expert scores for horses BCS 7–8 (*n* = 27)

Prior to receiving information and instructions on scoring, agreement with the expert’s judgement ranged from 0% for horses and ponies of BCS 3 and 4 to 60% for horses and ponies of BCS 6. After receiving information and instructions, agreement rose for horses and ponies of BCS 4 (from 0% to 33.33%), BCS 5 (from 30 to 40%) and BCS 8 (21% to 36%) however it fell for horses and ponies of BCS 6 (from 60 to 40%) (Fig. 3). Agreement between the expert and owners’ initial judgement was fair (κ = 0.311, *P* < 0.001) [22] to good (ICC = 0.502, *P* < 0.001) [23]. After the owners received information and instructions on body condition scoring and how to apply it to their horses and subsequently applied it to their horses, agreement rose slightly but was still fair (κ = 0.381, *P* < 0.001) [22] to good (ICC = 0.561, *P* < 0.001) [23]. Fig. 4.

### Thematic analysis

Owner responses to the open-ended questions regarding body condition and weight and feedback on the scoring system itself were explored separately. Initial codes were identified within these two sets of responses (Tables 1, 2, column 1). The codes were then grouped into themes with three themes identified in the body condition and weight responses and three in the feedback on the scoring system responses (Tables 1, 2, column 2). The themes were then reviewed by applying them to the owner responses. One theme was split into two in the body condition and weight responses whilst two themes in the feedback on the body condition scoring system responses were combined into one (Tables 1, 2, column 3). Finally, some of the themes were renamed to reflect the codes within them.

**Table 1:** Body condition and weight codes and themes

*Initial codes*	*Initial themes*	*Final themes*
Aware of possible weight issues & doing something about it	Awareness and feels in control	Awareness and feels in control
Just the way it/he/she is	Awareness but doesn't feel in control	Awareness but doesn't feel in control
Out of owner's control		
Knows something needs changing but excuses/doesn't know how/hasn't yet		
Input from others the owner trusts (yard, farrier, vet)	Other feelings/actions around weight	Seeks expert opinion
Doesn't trust own judgement		
Weight is lesser of two evils		Weight balanced against other factors
Horse is happy with current situation

**Table 2:** Feedback on the body condition scoring system codes and themes

*Initial codes*	*Initial themes*	*Final themes*
The system or parts of it are useable/useful	The system is useful	The system is useful
Owner never thought to look at multiple/certain body areas		
The system is too technical for owner to use/parts are too difficult	The system is too technical	The system could be improved
The system doesn't account for different breeds/body types/ages/sexes	The system could be improved	
Critical of particular parts of system		

### Thematic analysis of owner responses – body condition and weight

The analysis identified four themes with regards to owners’ perceptions of their horses’ body condition and weight. These were: Awareness of body condition issues but feels in control; Awareness of body condition issues and does not feel in control; Seeks expert opinion; Weight balanced against other factors.

The most commonly occurring theme was an awareness of body condition issues with a feeling of control. Owners mentioned being aware of weight issues, already utilizing solutions such as turnout on paddocks with less grass or changing forage type, and utilizing monitoring methods such as routine photos, weight tape or weigh scales use. Owner 35 stated that they “never let her get an apple bum” whilst Owner 31, whose horse has a history of laminitis and won a showing class two weeks before being diagnosed, stated that “his health is more important than a red ribbon” and reported taking multiple measures to address his weight.

The second most common theme to arise was that of an awareness of body condition issues but where the owner does not feel in control. Within this theme, owners expressed that body condition issues were out of their control, that they didn’t know how to address them, or that they were thinking of or wanted to utilize particular solutions but hadn’t. For example, Owner 6 stated that they were keeping an eye on their horse’s weight but that the horse appeared to be “gaining more than she’s eating”, while Owner 54 said that their horse jumps out of the field to access better grass. Some owners wondered or expressed that body condition issues were down to the horse’s age, type or height: “Ponies can be naturally tubby.” (Owner 10) while Owner 11 stated that they don’t know if it’s normal for their horse’s type and height for the ribs to be visible.

The seeking of advice from other individuals trusted by the owner, including yard managers, farriers and vets was the third theme identified with regards to owners’ perceptions of their horses’ body condition and weight. Farriers were mentioned most by owners under this theme with Owner 12 stating that their farrier said their horse was “very fat” and others saying that their farrier will tell them if their horses are getting fat. Comments in this theme also reflected an uncertainty about their own judgement from the owner with Owner 26 stating that they “might be one of those people who think their horse is thin” and Owner 47 wondering if their judgement of their horse as “scrawny” is only in comparison to ponies.

The final theme identified with regards to owners’ perceptions of their horses’ body condition and weight was that of balancing weight against other factors. Within this theme owners expressed opinions that issues with condition are the lesser of two evils or that they don’t think about condition. Owner 20 said that weight or condition monitoring “hadn’t crossed [their] mind.” Owner 14 expressed that “[horse] is so happy with field grass” while Owner 40 said they had considered using a grazing muzzle for weight but that it “seems cruel.”

### Thematic analysis of owner responses – feedback on the body condition scoring system

Two themes were identified within owners’ feedback regarding the body condition scoring system. The first of these was that the system was useful for assessing or monitoring body condition. Within this theme, owners expressed feedback that the scale, or parts of it, were useable or useful or that it included body areas they hadn’t considered assessing. There was also an element of the use of the system making the owners re-think their previous judgement; Owner 27 said that using the system made them look at their horse differently and Owner 14 that it made them “think twice” regarding their horse’s condition.

The second theme identified was that the scale could be improved. Within this theme, some owners were critical of specific parts of the scale, highlighted that it in their opinion it wasn’t applicable to all breeds, types, ages or sexes or suggested specific ways of improving the useability of the scale including the addition of illustrations or photos. Other owners felt that the scoring system is too technical or difficult to use, either in whole or in part, with some owners who expressed the “too technical” opinion stating that they were unfamiliar with some body parts which were being referenced (specifically tailhead, hook bones and loins); others expressed concern that there is no difference between descriptive terms such as “spongy” and “soft”, or that the different scores were too close together. Owner 60 stated that the scale needs to be “more idiot-proof.”

### Yard cultures

The expert scores and initial owner judgements from the three yards with the highest numbers of participating horse-owner combinations were examined (Table 3).

**Table 3:** Comparisons of equine BCS and owner judgements between three yards

Yard A			Yard B			Yard C		
*Horse ID*	*Expert BCS*	*Owner Judgement*	*Horse ID*	*Expert BCS*	*Owner Judgement*	*Horse ID*	*Expert BCS*	*Owner Judgement*
1	4	5	1	4	5	1	6	6
2	4	6	2	4	6	2	6	8
3	5	5	3	6	6	3	7	5
4	5	5	4	6	6	4	7	8
5	5	6	5	6	8	5	8	5
6	6	6				6	8	5
7	7	7						
8	8	9						

In Yard A, three quarters (75%) of horses surveyed were in appropriate condition (compared with 51.67% of all horses assessed), with 37.5% being in optimal condition (compared with 10% of all horses assessed). Owner accuracy in Yard A was 50% (accuracy of all participants was 18%). None of the owners in Yard A underestimated their horse’s condition however half overestimated by one point. Yard A participants’ horses consisted of mostly of high-level competition horses however two (one pony) were not competing and one of these was not being ridden at the time due to a diagnosis of gastric ulcers. Yard A had a weighing scale on site although not all owners reported using it, with some reporting using a weight tape or assessing visually or by how the horse feels when riding. All horses received different types and amounts of feed. The owners of horses 7 and 8 reported being aware of their horses’ need to lose weight. For those in work, exercise intensity ranged from 1 easy and 2–3 medium sessions per week to 1–2 easy, 4–5 medium and the occasional hard work session per week. Turnout for most horses was all day but was reduced for horses who required it for weight or other reasons. Participants in Yard A expressed comments within the “awareness and feels in control,” “weight balanced against other factors” and “awareness but doesn’t feel in control” themes, with the first of those themes being most prevalent among the owners in this group.

In Yard B, all horses were in appropriate condition, although none were in optimal condition. Owner accuracy was 40%. None of the participants in Yard B underestimated their horse’s condition however 60% overestimated by one or two points. Yard B participants’ horses were used for dressage, showing and showjumping if competing, although to a lower level than those in Yard A. Two owners in Yard B reported taking photos to monitor condition. All horses received different types, or combinations of types, and amounts of feed. Exercise intensity ranged from 2 easy and 2 medium sessions to 1–2 easy and 4–5 medium sessions per week Turnout arrangements differed between horses, ranging from no turnout to living out full time in summer. Participants in Yard B expressed comments within the “awareness and feels in control” and “seeks expert opinion” themes, with the first of those themes being more prevalent among the owners in this group.

In Yard C, two thirds of horses were over-conditioned, with one third being appropriately conditioned; none were in optimal condition. Owner accuracy in Yard C was 12.5%. Half of the owners in Yard C underestimated their horse’s condition by two or three points, with all believing their horses were in optimal condition, whilst a third underestimated their horse’s condition by one or two points. The horses and ponies in Yard C were used for pleasure riding with one being retired. None of Yard C’s owners reported using any specific means of monitoring their horses’ condition despite one mentioning that the yard owner uses a BCS scale. Most of the horses in Yard C were either fed a coarse mix or received no hard feed. Exercise intensity for the non-retired horses ranged from 1–2 easy and 1–2 medium sessions to 5 medium sessions per week. Two of the horses were stabled with the others living out. Participants in Yard C expressed comments within the “awareness but doesn’t feel in control,” “awareness and feels in control” and “seeks expert opinion” themes, with the first of those themes being most prevalent among the owners in this group.

## Discussion

In this study, 45% (*n* = 27) of horses were overweight or obese. Owners’ ability to accurately judge their horse’s condition was fair, with owners of overweight horses underestimating their condition and owners of underweight horses overestimating their condition. Providing owners with a descriptive scoring scale and instructions as how to carry out body condition scoring had very little effect on the accuracy of their judgements. Owners’ actions and feelings regarding monitoring and managing their horses’ condition were varied. Only a quarter of owners used some method of regularly monitoring their horse’s condition. Owners’ feedback on the utility and useability of the body condition scoring scale were mixed with an overall trend towards the concept of the system and specific parts of it being useful but that parts were too technical.

The number of horses who were overweight or obese in this study is similar to that found in studies in Scotland [4]. If this percentage is representative of the wider Irish horse population, nearly half are at increased risk of obesity-related disease. As opposed to another study [6] that found that height and supplementary forage feeding were both associated with obesity, no such associations were found in this study, nor were factors including turnout details, exercise, yard type or condition monitoring. The amount of forage fed was not tested as a variable in this study as owners would not have been able to give an accurate weight of forage fed.

Only 15 owners reported using a specific means (weight tapes, BCS, weighing scales or routine photos) of monitoring condition, although the majority did state that they would notice weight gain or loss in specific areas. What is notable is that the body area mentioned most by owners in this study was the belly, which is not assessed in either of the two BCS systems commonly used; however belly girth measurement has been found to be a reliable indicator of weight and weight loss [24, 25]. It may be that this factor could be a useful indicator of condition that owners seem to already be aware of, however caution would be needed before recommending it as a means of condition monitoring as some horses may have large belly girth measurements for other reasons, including muscular changes with age, high worm burdens or ingestion of large amounts of straw or poor-quality hay.

Similarly to a recent study of horse owners in Italy [7], agreement between the expert’s BCS score and the owners’ initial judgements was found to be fair. Also, as found in other studies [3, 6], owners of overweight or obese horses and ponies often underestimate their condition, however this study also found that owners of those that are underweight overestimated their condition, suggesting that there may be a tendency among owners to believe their horses are in or are close to optimal condition, regardless of whether they are over- or under-weight.

Body condition scoring may not be accurately useable by a significant proportion of horse owners, since agreement between the expert’s scores and the owners’ judgements was still classed as fair agreement despite the use of the scoring scale. A similar lack of change in accuracy has been seen in dog owners where the use of a BCS chart did not improve the accuracy of their judgement of their dogs’ condition, particularly that of overweight dogs [26]. This fact, combined with other research on perceptions of canine weight [10], as well as that of perceptions of human weight [11, 12] show that this phenomenon is by no means exclusive to horses and their owners but is perhaps just one example of a perceptual problem present across societies.

That a proportion of owners are aware of issues or potential issues with their horses’ body condition and that this awareness is accompanied by a feeling of control is a positive finding. There remain owners who have similar awareness but do not feel in control. Some of these view condition as an aspect of the horse that they cannot influence, for example an older horse being underweight because of its age or a pony being overweight because of its breed. The potentially obesogenic environment in which an owner keeps their horse (social and physical) [14] and potentially the need for owners to make the transition between seeing weight as an integral part of the horse’s shape to something that they can influence [9] are factors that may influence how successful education and encouragement could be. Health-related behaviour change among clients, whether for the sake of themselves or animals under their care, is however complex and factors including communication styles [27], workload pressures affecting time available during consultations [28], feelings of control or lack of same within the owner [29], entrenched attitudes and behaviours [30] and perceptions of professionals’ roles [28] may all present barriers to behaviour change.

The most challenging theme with regards to changing owner perceptions of overweight horses and ponies is likely that of not considering condition or weight to be important or as important as other factors. Previous research has identified that owners can perceive weight-loss measures, such as less food and/or more exercise, as negatives in terms of welfare [9].

Our findings would suggest that Henneke system is not a reliably accessible body condition scoring tool for owners to use. The lack of significant improvement in the accuracy of owners’ judgement of their horses’ condition after receiving information on and instructions as to the use of the Henneke BCS system supports this conclusion. It is possible though, given the problems that appear to be common in the use of subjective scales such as BCS scales by horse and pet owners [3, 4, 6, 26] that the future lies in more objective measurements of condition. While research has identified a number of possible alternative methods of classifying horses as overweight or obese, including ultrasonography [31] and morphometrics [24], as this paper has been focused on the perceptions and classification of condition by horse owners, those that have the potential to be easily and accurately utilized by owners are of most interest. The most promising measurement in this regard may be the formula derived using height, heart girth, belly girth and neck circumference [32].

The identification of potential yard cultures was a notable outcome of this study. Previous research [9, 14] has highlighted the fact that yards, in the sense of both the physical environment and influence on the horse owner of the people in it, can create an obesogenic environment for the horses in them. The notable differences between the yards were in the owners’ behaviour and views with regards to condition monitoring; those in Yards A and B were more accurate in their judgements of their horses’ condition than those in Yard C where most of the horses were overweight. Whilst further research would be required to investigate the effects of the yard on rates of obesity and the ability of owners to accurately judge their horses’ condition, it is important to be aware that the yard and the influence of an owner’s peers and how they monitor and in what condition they maintain their horses may play a role in the presence or absence of obesity in an individual horse. Peers or societal norms have been shown to play a role in the influence of human behaviour specifically in the management of horses [14], but also in other areas such as the adoption of organic farming practices [33] or approach to parasite control in bovine herds [34].

There were some limitations to this study. The actual BCS of each horse was determined by a single equine vet; it may have been better to have had multiple equine vets score each horse and use a consensus score. Certain criteria, such as exercise and amount fed, may have been under- or over-estimated by the owners in giving their answers. For owners with more than one horse, given the inclusion of the clinical exam, they may have been more likely to choose one with a history of poorer health or that they had particular concerns regarding. While the self-selecting nature of the participants ensured no bias from the researchers, it did result in far fewer underweight horses included than overweight horses. Finally, it is possible that training from the expert in how to perform the body condition scoring would have elicited better results in the post-instruction scoring however the researchers elected not to do this as it is not a feasible real-world solution.

## Conclusions

The results of this study, both qualitative and quantitative, strongly suggest that the use of body condition scoring by owners does not elicit accurate assessments of their horses’ condition. Further research, into alternative methods of equine body condition assessment by owners is warranted. Rendle et al. (2018) suggest that “[i]n the future, a combination of objective measurements may prove to be easier and more robust to apply than the BCS system for owners” however they state that more work would be required for validation, which is still the case. The high proportion of horses and ponies that were identified as overweight or obese, and the health consequences that brings, makes this a matter of some urgency. In the meantime, responsibility for identifying overweight horses, making sure owners are aware of the risks and enabling them in making changes, perhaps using the specific resources mentioned above, to facilitate weight loss remains with equine veterinarians, nurses and other professionals.

## Supplementary Information

**Additional file 1.**

**Additional file 2.**

## Data Availability

The datasets analysed during the current study are available from the corresponding author on reasonable request.

## Abbreviation

BCS: Body Condition Score
