# Supplementary material for: Rate of obesity within a mixed-breed group of horses in Ireland and their owners’ perceptions of body condition and useability of an equine body condition scoring scale
Source: Ir Vet J. 2023 Apr 6;76:9. doi: 10.1186/s13620-023-00237-w (PMC10077657; doi:10.1186/s13620-023-00237-w)
Supplement: Supplementary file 1 — Additional file 1. [file 13620_2023_237_MOESM1_ESM.pdf]

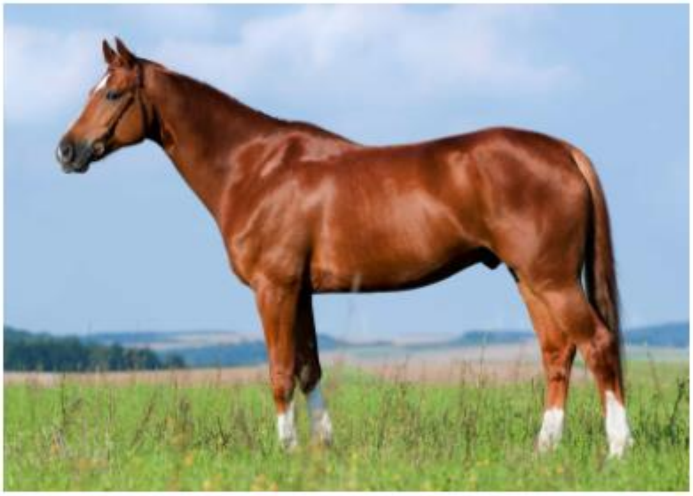

Many athletic horses are kept at a BCS of 5, sometimes 6, depending on their discipline. Some equine athletes, such as endurance horses, will have condition scores between 4 and 5. | Photo: iStock

By Fernanda Camargo, DVM, PhD; Laurie Lawrence, PhD; and Bob Coleman, MS, PhD, PAS, of the University of Kentucky Department of Animal and Food Sciences

As we understand more about the impact that obesity and emaciation have on animal health, it is imperative that we strive to keep our horses at an optimum body condition. Since 1983, a procedure developed by Don Henneke, PhD, has served to provide a standard body condition scoring system that can be used across breeds and by all horse people. The system assigns a numerical score—1 through 9—based on the amount of fat that has accumulated in the important areas used to assess horses' body condition.

The Body Condition Scoring System

The Henneke system assesses accumulated fat both visually and by palpation in each of six areas: ribs, behind the shoulder, withers, loin, tailhead, and neck. A numerical value is assigned based on the fat accumulated in all six areas (Table 1).

Table 1. Henneke Body Condition Scoring System.

Condition	Ribs	Neck	Withers	Loin	Tailhead	Shoulder
1: Poor	Tailhead (pinbone) and hook bones project prominently	Bone structure easily noticeable; animal extremely emaciated; no fatty tissue can be felt	Bone structure easily noticeable	Spinous processes project prominently	Spinous processes project prominently	Bone structure easily noticeable
2: Very Thin	Slight fat cover over ribs; ribs easily discernible	Faintly discernible; animal emaciated	Faintly discernible	Slight fat covering over base of spinous processes; transverse processes of lumbar vertebrae feel rounded; spinous processes are prominent.	Tailhead prominent	Shoulder accentuated
3: Thin	Slight fat cover over ribs; ribs easily discernible	Neck accentuated	Withers accentuate	Fat buildup halfway on spinous processes but easily discernible; transverse processes cannot be felt	Tailhead prominent but individual vertebrae cannot be visually identified; hook bones appear rounded but are easily discernible; pin bones not distinguishable	Shoulder accentuated
4: Moderately Thin	Faint outline discernible	Neck not obviously thin	Withers not obviously thin	Negative crease along back	Prominence depends on conformation; fat can be felt; hook bones not discernible	Shoulder not obviously thin
5: Moderate	Ribs cannot be visually distinguished, but can be easily felt	Neck blends smoothly into body	Withers rounded over spinous processes	Back level	Fat around tailhead beginning to feel spongy	Shoulder blends smoothly into body
6: Moderately Fleshy	Fat over ribs feels spongy	Fat beginning to be deposited	Fat beginning to be deposited	May have slight positive crease down back	Fat around tailhead feels soft	Fat beginning to be deposited
7: Fleshy	Individual ribs can be felt, but noticeable filling between ribs with fat	Fat deposited along neck	Fat deposited along withers	May have positive crease down back	Fat around tailhead is soft	Fat deposited behind shoulder
8: Fat	Difficult to feel ribs	Noticeable thickening of neck; fat deposited along inner buttocks	Area along withers filled with fat	Positive crease down back	Tailhead fat very soft	Area behind shoulder filled in flush with body
9: Extremely Fat	Patchy fat appearing over ribs	Bulging fat; fat along inner buttocks may rub together; flank filled in flush	Bulging fat	Obvious positive crease down back	Building fat around tailhead	Bulging fat

Adapted from Henneke et al. Equine Vet J. (1983) 15 (4), 371-372

Ribs

The first place to look when assessing a horse's body condition score (BCS) is the ribcage. If ribs are easily seen, the horse will have a score over the ribcage below a 5. If you cannot see the ribs, then the score should be a 5 or above. During winter and spring it might be difficult to see ribs because of the horse's coat, so it is always important to run your fingers across the ribcage to assign the correct score.

A very thin horse will have prominent ribs—easily seen and felt—with no fat padding. As the horse gains weight and body condition, a little padding can be felt around the ribs. By score 5, the ribs will no longer be visible, but can be easily felt. Once the body condition score is above 7, the ribs become more difficult to feel.

Shoulder

A BCS of 5 means the shoulder blends smoothly with the body. At increasing condition scores, fat is deposited behind the shoulder and becomes bulging. This observation is especially true in the region behind the elbow. The shoulder's bony structures will become more visible as the scores drop below 5.

Tailhead

In a very thin horse, the tailhead is prominent and easily discernible. Once the horse starts gaining weight, fat fills in around the tailhead. As the condition score exceeds 7, the fat will feel soft and begin to bulge.

Withers

If a horse is very thin, no fat will be deposited between the top of the shoulder blade and the spinal vertebrae, making the two structures easily discernible. As the horse's condition score increases, fat fills in between the top of the shoulder blade and spinal vertebrae; so, at a condition score of 5, the withers will appear rounded. As horses approach the high end of the condition scoring scale, the withers will be bulging with fat.

Neck

In a very thin horse, you might be able to see the neck's bony structures. As the horse gains condition, fat will be deposited on the top of the neck. At a condition score of 5, the neck blends smoothly into the body. Body condition scores of 8 and 9 are characterized by a neck that is thick all around with fat evident at the crest.

Loin

The loin is the area of the back just behind where the saddle sits. At a condition score of 5, the loin area will be relatively level—the spine is not sticking up, nor is there a dent or crease along the spine. At condition scores below 5, the spine starts to become prominent; this is sometimes called a "negative crease." A very thin horse will have an obvious ridge down the back where the vertebrae of the spine become obvious. As the condition score increases above a 5, fat begins to build up on either side of the spine and a visible crease starts to appear.

Overall Score

After each area is assessed and assigned a score (not all horses will get the same score at each location) you can average all the scores to get to a final overall score. For example, a horse might score 6 on some areas and 7 on others.

This information sheet consists of extracts from: Body Condition Scoring Horses: Step-by-Step, posted on thehorse.com on 15th January 2019 by University of Kentucky College of Agriculture, Food & Environment. https://thehorse.com/164978/body-condition-scoring-horses-step-by-step/ (accessed 24/06/2021).
