# Supplementary figures and images for: Rate of obesity within a mixed-breed group of horses in Ireland and their owners’ perceptions of body condition and useability of an equine body condition scoring scale
Source: Ir Vet J. 2023 Apr 6;76:9. doi: 10.1186/s13620-023-00237-w (PMC10077657; doi:10.1186/s13620-023-00237-w)

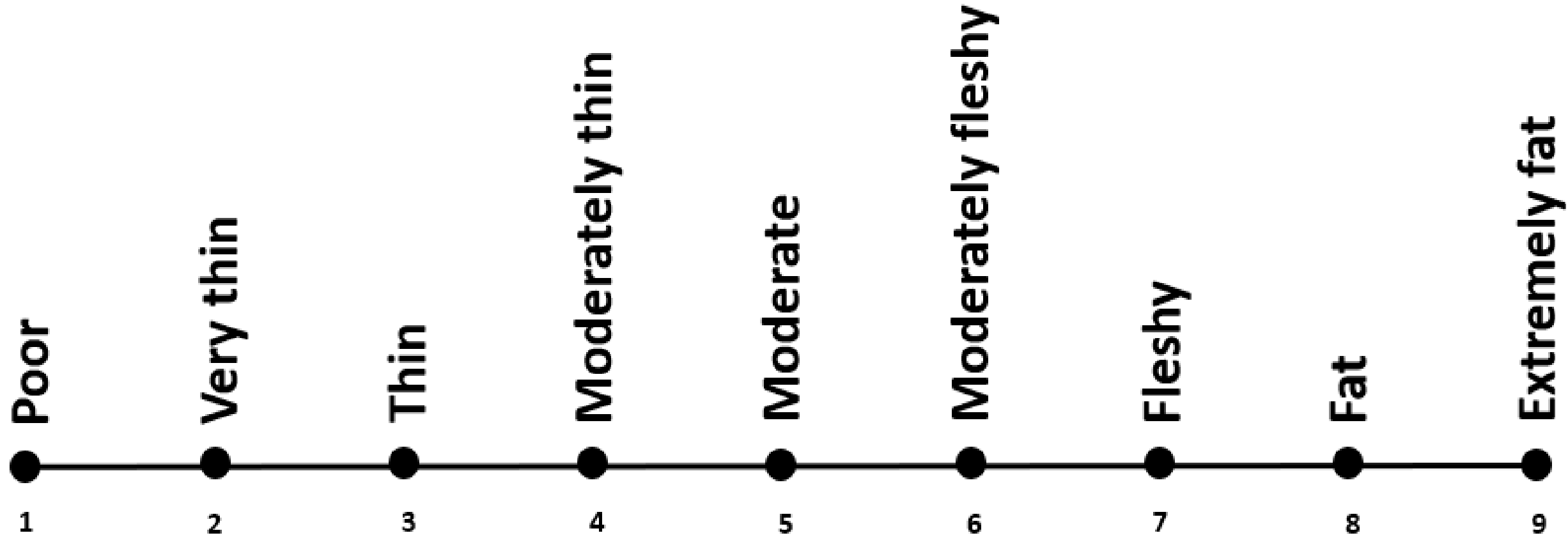

Supplement: Supplementary file 2 — Additional file 2. [file 13620_2023_237_MOESM2_ESM.pdf]
